# 2-Phenyl-3-(Phenylselanyl)Benzofuran As a Promising Antidepressant Candidate: Mechanistic Insights Into Nitrergic Modulation and Subchronic Efficacy and Safety Profiling

**DOI:** 10.1007/s12035-026-06058-6

**Published:** 2026-07-16

**Authors:** Taís da Silva Teixeira Rech, Ediandra Tissot Castro, Mariana Parron Paim, Dianer Nornberg Strelow, José Sebastião Santos Neto, Gustavo Bierhals Blödorn, Diego Alves, Suzan Gonçalves Rosa, César Augusto Brüning, Cristiani Folharini Bortolatto

**Affiliations:** 1 Programa de Pós-Graduação em Bioquímica e Bioprospecção, Laboratório de Bioquímica e Neurofarmacologia Molecular (LABIONEM), Centro de Ciências Químicas, Farmacêuticas e de Alimentos (CCQFA), Universidade Federal de Pelotas (UFPel), CEP 96010-900 Pelotas, RS Brasil; 2 Instituto de Química, Universidade Federal de Goiás (UFG), Goiânia , GO CEP 74690‑900 Brasil; 3 Universidade Federal do Pampa (UNIPAMPA), CEP 97500-970 Uruguaiana, RS Brasil; 4 Programa de Pós-Graduação em Química (PPGQ), Laboratório de Síntese Orgânica Limpa (LASOL), Centro de Ciências Químicas, Farmacêuticas e de Alimentos (CCQFA), Universidade Federal de Pelotas (UFPel), CEP 96010-900 Pelotas, RS Brasil

**Keywords:** SeBZF**1**, Antidepressant, NO-cGMP, Mice, Selenium, Tail suspension test

## Abstract

The compound 2-phenyl-3-(phenylselanyl)benzofuran (SeBZF**1**), a selenium-containing molecule based on a benzofuran scaffold, has shown antidepressant-like effects. This study investigated the involvement of the nitric oxide (NO)-cyclic guanosine monophosphate (cGMP) pathway, the efficacy of subchronic treatment in male Swiss mice, and computational analyses. Acute SeBZF**1** administration (50 mg/kg, i.g.) showed antidepressant-like effects in the tail suspension test (TST). These effects were reversed by pretreatment with L-arginine (a NO precursor, 750 mg/kg, intraperitoneal, i.p.) or sildenafil (a phosphodiesterase-5 inhibitor, 5 mg/kg, i.p.), suggesting NO-cGMP pathway involvement. A subeffective dose of SeBZF**1** (1 mg/kg, i.g.) elicited synergistic antidepressant-like effects when combined with N(ω)-nitro-L-arginine methyl ester (L-NAME, a nitric oxide synthase (NOS) inhibitor, 10 mg/kg, i.p.) or 7-nitroindazole (a specific neuronal NOS inhibitor, 30 mg/kg, i.p.). Furthermore, methylene blue (an inhibitor of NOS and soluble guanylate cyclase (sGC), 10 mg/kg, i.p.) and 1H-[1,2,4]Oxadiazolo[4,3-a]quinoxalin-1-one (ODQ, a sGC inhibitor, 30 pmol/site, intracerebroventricular), both soluble guanylate cyclase (sGC) inhibitors, enhanced the subeffective SeBZF**1** dose. Subchronic SeBZF**1** treatment (1 mg/kg, i.g., for 35 days) significantly produced antidepressant-like action without causing weight loss or organ damage. Biochemical analysis revealed reduced nitrate/nitrite levels in the prefrontal cortex and hippocampus, indicating modulation of NO signaling. Molecular docking suggested interactions between SeBZF**1** and NOS isoforms, while pharmacokinetic analysis suggested brain penetration and absence of overt toxicity. The present study concludes that SeBZF**1** exhibits antidepressant-like effects through the NO–cGMP pathway, is effective at low subchronic doses, and displays favorable pharmacokinetic and safety profiles, supporting its potential as a novel antidepressant candidate.

**Supplementary Information:**

The online version contains supplementary material available at 10.1007/s12035-026-06058-6.

## Introduction

Depression is a disease characterized by significant detrimental effects on mood and behavior, with an increasing global prevalence. It is currently one of the most common conditions affecting the central nervous system (CNS) [1]. Depression involves symptoms such as persistent sadness, anxiety, irritability, insomnia, and difficulty concentrating [2]. Most prescribed antidepressants act through monoamine regulation; however, many fail to produce adequate effects and often cause adverse side effects. Moreover, the remission rates remain low [3, 4]. Given these limitations, developing safer and more effective treatments with multi-target potential may offer improved therapeutic outcomes and better management of depression.

Findings from previous studies have demonstrated the role of the nitrergic system in the pathophysiology of mood disorders, including depression [5, 6]. The nitric oxide (NO)/cyclic guanosine monophosphate (cGMP) pathway is involved in regulating various behavioral and emotional functions [7]. NO is synthesized in the brain from L-arginine (L-ARG) by nitric oxide synthases (NOS) and modulates synaptic transmission through the activation of soluble guanylate cyclase (sGC), thereby increasing cGMP levels [5]. NO mediates several neurobiological functions related to depression, as NOS inhibition has been reported to exert antidepressant-like properties. Thus, the inhibition of NO synthesis and the consequent decrease in cGMP concentration can produce antidepressant-like effects [8–10].

A dysfunctional nitrergic system leads to an imbalance in NO metabolite levels, with increased concentrations being associated with depression and nitrosative stress [11]. Elevated NO levels are also related to neuronal death due to excitotoxicity, caused by the excessive release of glutamate and overstimulation of postsynaptic glutamate receptors [12]. Thus, although the involvement of the NO–cGMP pathway in antidepressant-like effects is well established, investigating how novel compounds modulate this signaling cascade remains essential for understanding their specific mechanisms of action.

Selenium compounds and benzofuran derivatives have demonstrated consistent antidepressant-like effects in previous studies [13–15]. Se-based compounds may act by modulating the nitrergic pathway [16–18]. In this context, 2-phenyl-3-(phenylselanyl)benzofuran (SeBZF**1**) is an organic selenium compound containing a benzofuran scaffold that exhibits antidepressant-like activity in both male and female mice. SeBZF**1** shows antidepressant-like action modulated by serotonergic (5-HT_1A_ and 5-HT_2A/C_) [19], dopaminergic (D_1_ and D_2_) [20], and glutamatergic (NMDA, AMPA, and kainate) pathways [21], as well as by the modulation of the enzyme monoamine oxidase (MAO) [22]. Furthermore, SeBZF**1** has not shown toxic effects [19, 20]. Despite the evidence regarding the antidepressant-like effects of SeBZF**1** and its modulation of monoaminergic and glutamatergic systems, its potential interaction with the nitrergic pathway has not yet been investigated. Importantly, understanding whether this pathway contributes to SeBZF**1** activity is relevant not only to identify an additional target, but to provide a more integrated view of its mechanism of action.

In this context, the present study aimed to characterize the involvement of the nitrergic signaling pathway in the antidepressant-like effects of SeBZF**1** using pharmacological, biochemical and computational approaches. In addition, we evaluated the antidepressant-like effects of the compound following subchronic treatment and performed computational analyses to predict its pharmacokinetic profile.

## Materials and Methods

### Animals

This study was conducted using male Swiss mice obtained from a local breeding colony. The mice weighed between 25–35 g (approximately 6–7 weeks old), and were housed in a separate animal room under controlled conditions (22 ± 1 °C, 12-h light/12-h dark cycle lights on at 7:00 a.m.) with free access to water and standardized pelleted food. The animals were habituated for at least 1 h before the beginning of the tests. All experimental procedures were approved by the Ethical Committee on Animal Experimentation of the Federal University of Pelotas (UFPel) (protocol no. 14064–2019), Brazil, affiliated with the National Council for the Control of Animal Experimentation (CONCEA), and were conducted in compliance with the National Institutes of Health Guide for the Care and Use of Laboratory Animals. This study was reported in compliance with the ARRIVE 2.0 guidelines. Every effort was made to guarantee the well-being of the animals.

### Chemicals

The compound SeBZF**1** was synthesized and characterized in the Laboratory of Sustainable Organic Synthesis and Technologies (SustenSin), Federal University of Goiás (UFG), using a previously reported method [23]. To confirm the structure of, the technique of Nuclear Magnetic Resonance (NMR) of carbon (^13^C) and hydrogen (^1^H) was used, proving that the synthesis was effective. The method of gas chromatography combined with mass spectrometry (GC/MS) was used to determine SeBZF**1**'s chemical purity (99.9%). The compound was dissolved in canola oil and administered via the intragastric (i.g.) route. Canola oil was used as the vehicle due to the lipophilic nature of SeBZF**1**, allowing appropriate solubilization for oral administration [24, 25]. The subeffective (1 mg/kg) and effective (50 mg/kg) doses of SeBZF**1** were selected based on a previous study [19] that established a dose–response relationship in male mice.

All the other chemicals were obtained from Sigma-Aldrich (St. Louis, MO, USA) or other standard commercial supplies. The substances methylene blue, sildenafil, L-nitroarginine methyl ester (L-NAME), L-ARG, and 7-nitroindazole (7-NI) were dissolved in saline solution and administered intraperitoneally (i.p.). Drug administration (i.p. or i.g.) was performed at a constant volume of 10 ml/kg body weight. 1H-[1, 2, 4]oxadiazole[4,3-a]quinoxalin-1-one (ODQ) was dissolved in saline solution containing 1% dimethyl sulfoxide (DMSO) and administered by intracerebroventricular (i.c.v.) route in a volume of 5 μl/site.

### Behavioral Tests and the Intracerebroventricular Injection Method

For behavioral tests, animals were randomly assigned to the experimental groups (n = 8–12 mice/group) and used only once. Subsets of each experimental set were replicated on different days during the same shift (8:00–11:00 a.m.). The apparatuses were cleaned with a 20% alcohol solution after each test sessions to remove the odor cues. The open-field test (OFT) and the tail suspension test (TST) were used for behavioral assessments. Sessions were recorded by camera for further analysis. Behavioral assessments were conducted by experimenters blinded to the treatment groups.

#### Open-field Test (OFT)

Locomotor and exploratory activities were assessed in the OFT to rule any motor abnormalities caused by the treatment. The open-field box (30 × 40 × 40 cm) was made of plywood, and the floor was divided into 9 equal zones. The OFT was performed immediately before TST [26]. Each animal was individually placed at the center of the apparatus, and the total number of crossings and rearings was recorded for 4 min.

#### Tail Suspension Test (TST)

The TST assesses depressive-like behavior, based on the assumption that reductions in immobility reflect the antidepressant-like effects of therapeutic interventions [27, 30]. The method consists of suspending the mouse by its tail, 50 cm above the floor, using adhesive tape placed approximately 1 cm from the tip [28]. The test was performed in an acoustically and visually isolated room. Total immobility time (sec), defined as the absence of scape-oriented movements, and the latency to the first immobility episode (sec) were manually recorded for 6 min.

#### Intracerebroventricular (i.c.v.) Injection

The i.c.v. administration was performed under isoflurane inhalatory anesthesia (3–5%) [29] according to a previously described procedure and by a trained researcher [30, 31]. Mice were placed in a chamber containing isoflurane, and once anesthesia was achieved, the injection was initiated immediately. The skull was punctured perpendicularly by a needle cannula (26G) that was connected to a 25 μl-Hamilton syringe through a probe. Coordinates were used for injections: 0.8 mm posterior to the bregma, 1.0 mm lateral to the sagittal suture, and 3.0 mm below the surface of the brain. Sterile saline (vehicle) or ODQ dissolved in sterile saline was injected in a volume of 5 μl. To prevent the injected solution from refluxing, the injection was administered over a period of 30 s, and the needle stayed in the body for an additional 30 s.

### Experimental Design

**Fig. 1:**
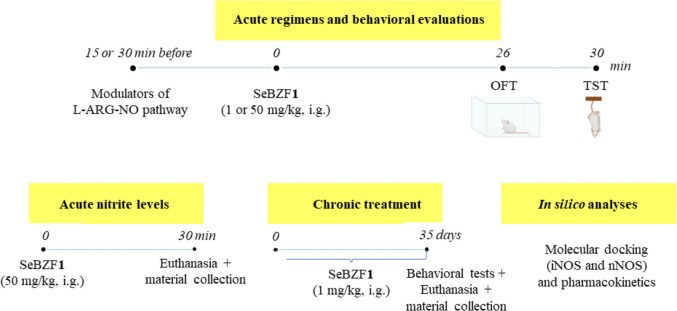
Experimental design

The complete experimental design of this study is illustrated in Fig. 1.

Behavioral experiments involving the co-administration of drugs capable of modulating the NO-cGMP pathway and SeBZF**1**, at effective or subeffective doses, were conducted. In all behavioral experiments, the OFT was evaluated 4 min before TST. Doses and pretreatment times were based on the scientific literature [9, 19, 32, 33].

#### Evaluation of the Possible Involvement of NO Pathway in the Acute Antidepressant-like Effects of SeBZF1

**Fig. 2:**
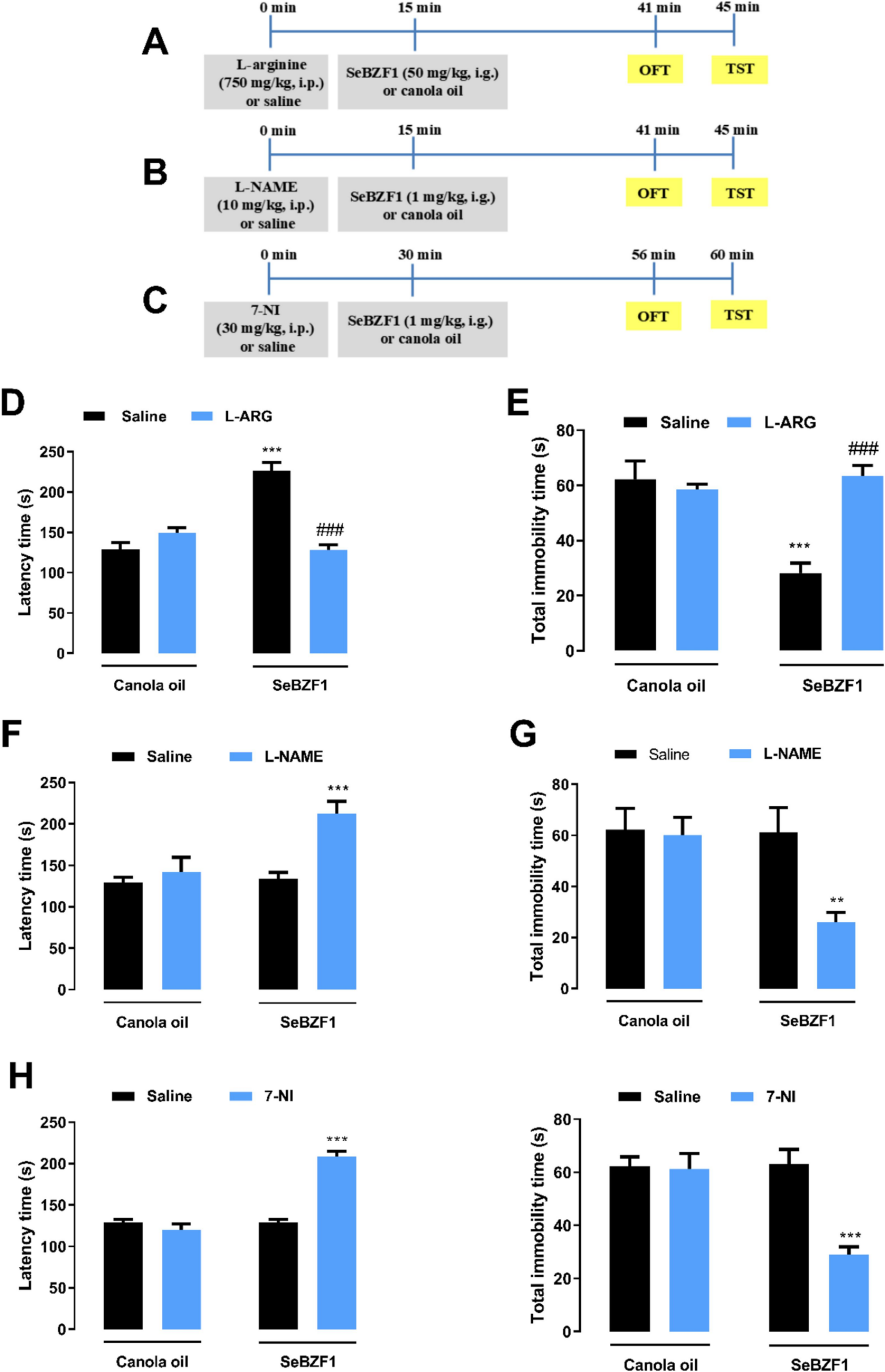
Evaluation of the involvement of L-ARG-NO pathway in the acute antidepressant-like action of SeBZF**1** in the mouse TST. Experimental design (**A**) and the effects of the pretreatment with L-ARG (750 mg/kg, i.p., a precursor in NO formation) on the anti-immobility effects of SeBZF**1** (50 mg/kg,i.g., an effective dose) is shown in panels D and E. Experimental design (**B**) and the effects of L-NAME (10 mg/kg, i.p., a non-selective NOS inhibitor) in combination with a subeffective dose of SeBZF**1** (1 mg/kg,,i.g.) is depicted in panels F and G. Experimental design (**C**) and the effects of 7-NI (30 mg/kg, i.p., a nNOS inhibitor) in combination with a subeffective dose of SeBZF**1** (1 mg/kg, i.g.) is demonstrated in panels H and I. Values are expressed as the mean ± S.E.M. (n = 8–12 mice/group). ^**^p < 0.01 and ^***^p < 0.001 compared with the control group; ^###^p < 0.001 and compared with the SeBZF**1** group as determined by two-way ANOVA followed by Tukey’s test

To investigate the relationship between the L-ARG-NO pathway and the antidepressant-like effects of SeBZF**1** on immobility time in the TST, we examined the effect of co-treatment of L-ARG, a substrate for NOS, with an effective dose of SeBZF**1**. Fifteen minutes after L-ARG injection (750 mg/kg, i.p.) [9], SeBZF**1** (50 mg/kg, i.g.) or its vehicle (canola oil) was administered. Thirty minutes later compound administration, the animals were evaluated by TST (Fig. 2A).

To investigate the role of the NO pathway in the antidepressant-like effects of SeBZF**1** by a possible inhibition of the NOS enzyme, mice were pretreated with L-NAME (10 mg/kg, i.p., a non-selective NOS inhibitor) [9] or 7-NI (30 mg/kg, i.p., a selective neuronal NOS inhibitor) [32]. Fifteen minutes after, L-NAME pretreatment, animals received SeBZF**1** (1 mg/kg, i.g., a subeffective dose) or vehicle (Fig. 2B). SeBZF**1** was administered 30 min after 7-NI pretreatment, and animals were evaluated 30 min later in the TST (Fig. 2C).

#### Evaluation of the Possible Involvement of cGMP in the Acute Antidepressant-like Effects of SeBZF1

**Fig. 3:**
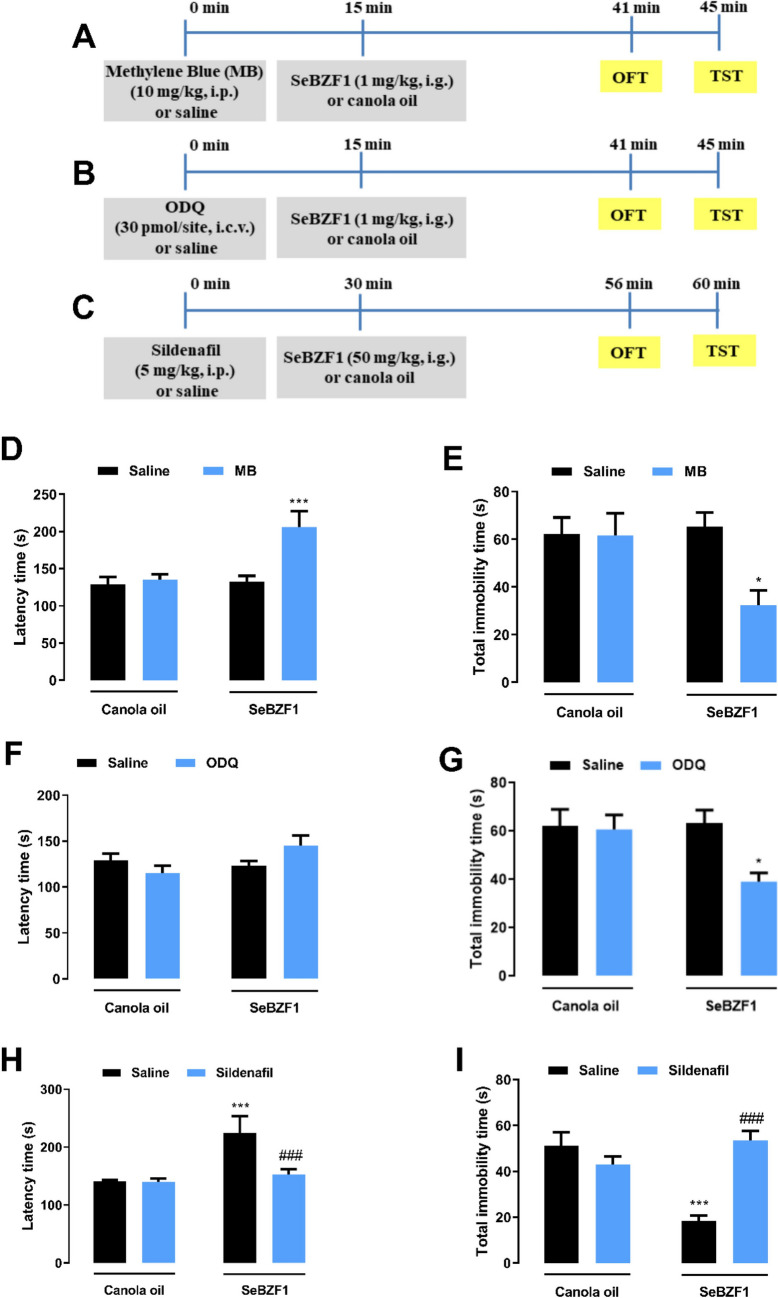
Evaluation of the involvement of the cGMP pathway in the acute SeBZF**1** antidepressant-like action in the mouse TST. Experimental design (**A**) and the effects of methylene blue (10 mg/kg, i.p., MB, an inhibitor of NOS and sGC) in combination with a subeffective dose of SeBZF**1** (1 mg/kg, i.g.) on mobility of mice are shown in panels D and E. Experimental design (**B**) and the effects of ODQ (30 pmol/site, i.c.v., a sGC inhibitor) in combination with a subeffective dose of SeBZF**1** (1 mg/kg, i.g.) are demonstrated in panels F and G. Experimental design (**C**) and the effects of pretreatment with sildenafil (5 mg/kg, i.p., a selective PDE-5 inhibitor) on the SeBZF**1** antidepressant-like action (50 mg/kg, i.g.) are depicted in panels H and I. Values are expressed as the mean ± S.E.M. (n = 8–10 mice/group). ^*^p < 0.05 and ^***^p < 0.001 compared with the control group; ^###^p < 0.001 compared with the SeBZF**1** group as determined by two-way ANOVA followed by Tukey’s test

To analyze the role of cGMP in SeBZF**1** antidepressant-like effects, a subeffective dose of the compound (1 mg/kg, i.g.) was co-administered with methylene blue (10 mg/kg, i.p., an inhibitor of NOS and sGC) [33]. Methylene blue was injected 15 min before the compound. Thirty minutes after SeBZF**1** treatment, the TST was conducted (Fig. 3A).

The combined effects of SeBZF**1** (1 mg/kg, i.g.) with ODQ (30 pmol/site, i.c.v., an sGC inhibitor) [33] were also investigated. ODQ was administered 15 min before SeBZF**1**, and the TST was conducted 30 min after SeBZF**1** treatment (Fig. 3B).

Additionally, the effects of a phosphodiesterase 5 (PDE-5) inhibitor, sildenafil, were evaluated. Mice were pretreated with sildenafil (5 mg/kg, i.p.) [32] or vehicle. Thirty minutes later, SeBZF**1** (50 mg/kg, i.g.), or vehicle was administered. Thirty minutes after SeBZF**1** treatment, the animals were evaluated in the TST (Fig. 3C).

#### Assessment of the Antidepressant-like Effects Following Subchronic SeBZF1 Treatment in Mice

To assess the antidepressant-like effects of the SeBZF**1** compound administered orally over an extended period, animals underwent a 35-day treatment protocol [34, 35]. Male mice (n = 10 animals/group) were randomly assigned to control and SeBZF**1** groups. The control group received canola oil, while the SeBZF**1** group received the compound orally at 1 mg/kg daily. Twenty-four hours after the last administration, animals were tested in the OFT and TST.

**Fig. 4:**
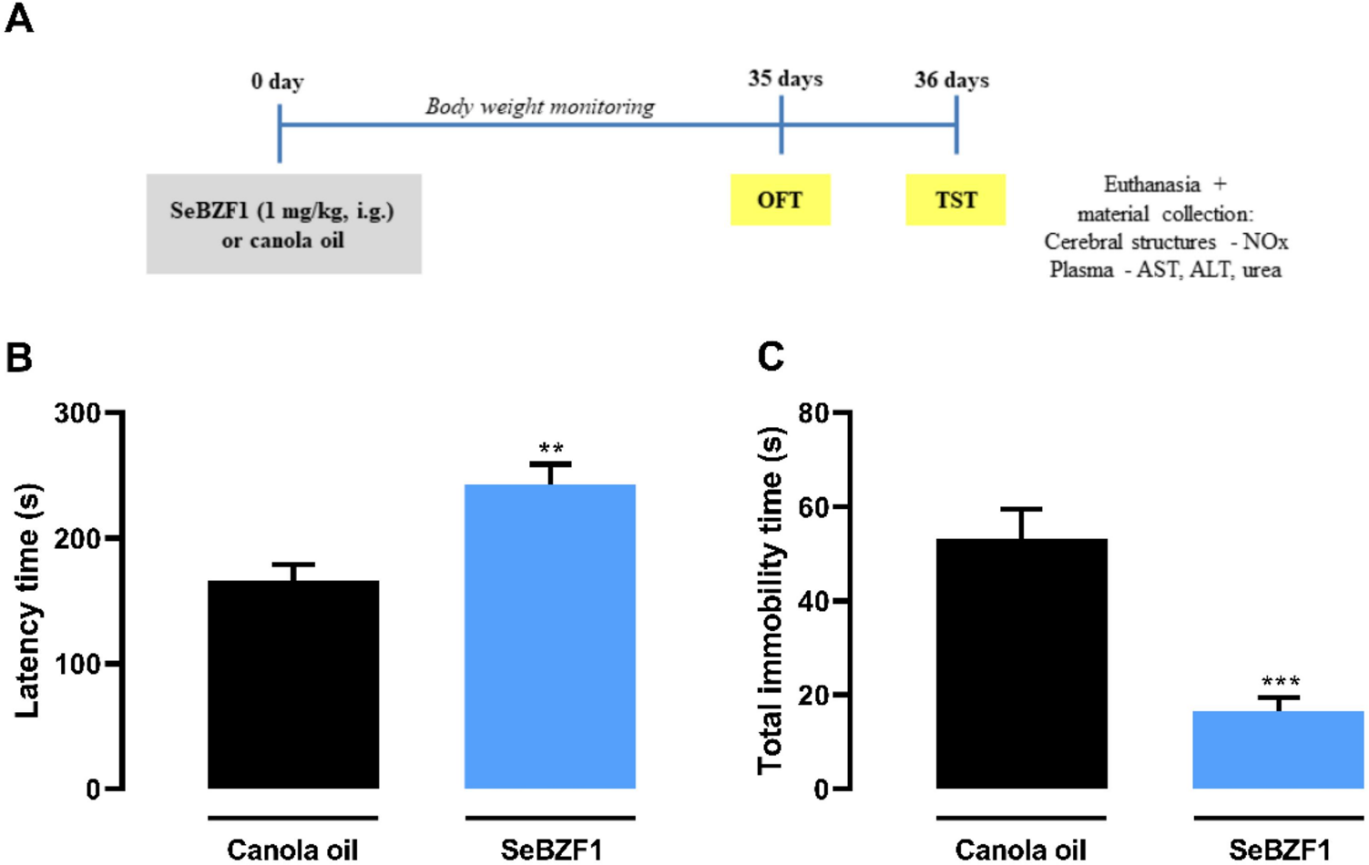
Impact of subchronic treatment with a low dose of SeBZF**1** (1 mg/kg, i.g.) on depressive-like behavior of male Swiss mice in the TST. The experimental design is illustrated in Fig.  4 A, while the latency time is presented in Fig. 4B and the total immobility time in Fig.  4 C (n = 10 mice/group). Values are expressed as the mean ± S.E.M. **p < 0.01 and ***p < 0.001 compared with the control group (canola oil). An unpaired *t* test was used for group comparisons

Additionally, alterations in NO levels following subchronic SeBZF**1** administration were investigated (Fig. 4A). Anesthesia was induced with an isoflurane overdose, and brain regions (hippocampus and prefrontal cortex) and blood samples were collected via cardiac puncture. Finally, to assess whether prolonged treatment with the compound SeBZF**1** would affect the body weight gain of the animals, they were weighed daily over 35 days of treatment, and the difference between the final weight and the initial weight was calculated.

#### Plasma Biochemical Tests for Toxicity Assessment

Concurrently, an evaluation was conducted to detect potential renal or hepatic toxicity associated with subchronic SeBZF**1** treatment. In summary, the activity of alanine aminotransferase enzymes (ALT), aspartate aminotransferase (AST), and urea levels were measured in the plasma of animals. For this, the animals were anesthetized with isoflurane for blood collection via cardiac puncture. Plasma was obtained by centrifuging the blood for 10 min at 700 g. All analyses were performed using commercial Bioclin® kits.

#### Evaluation of Cerebral Nitrite/Nitrate (NO_x_) Levels After Acute Treatment with SeBZF1

With the aim of investigating the effects of acute administration of SeBZF**1** (50 mg/kg, i.g.) on NO_x_ levels, an experiment was conducted. The animals received the compound and, after 30 min, were euthanized for collection the hippocampus and prefrontal cortex; to carry out ex vivo analyses of NO_x_ levels (n = 8–9 animals/group) [36].

#### Nitrite/nitrate (NO_x_) Level Assay

The hippocampus and prefrontal cortex were homogenized using 500 μl of zinc sulfate (ZnSO_4_ 200 mM) and 500 μl of acetonitrile (96%). The homogenate was centrifuged at 13,000 g for 30 min at 4 °C, and the supernatant was collected for analysis. The determination of NO_x_ content was performed using a reaction with 2% vanadium chloride (VCl_3_) (in 5% HCl), 0.1% N-(1-naphthyl) ethylenediamine dihydrochloride (NED), and 2% sulfanilamide (in 5% HCl). After incubating at 37 °C for 60 min, the absorbance was measured spectrophotometrically at 540 nm. The analysis was carried out to measure indirect NO levels, based on the reduction of nitrate to nitrite by VCl_3_ [36]. Nitrite/nitrate levels are expressed in nmol NO_x_/g tissue.

### Computational Analyses

#### Molecular Docking

The molecular docking tool was used to further our knowledge of the mechanism of action of SeBZF**1**. To prepare the compound for analysis, they were drawn using ChemDraw Ultra 12.0 software, and their geometries were optimized by software Avogadro 0.9.4. through UFF molecular mechanics force field calculations [37]. For protein preparation, the X-ray crystal structures of iNOS (PDB code 3E7G) [38] and nNOS (PDB code 6AV2) [39] were obtained from the Protein Data Bank (PDB) (http://www.pdb.org/). Positive controls were included for iNOS and nNOS, namely 1400 W (PDB code 1433) and 7-NI) (PDB code 1893), respectively [40]. The CHIMERA 1.5.3 software was employed for the removal of ligands [41], and Auto Dock Tools 1.5.6 (MGL; Molecular Graphic Laboratory) was used to eliminate ions and water molecules. Additionally, it was utilized to add polar hydrogens and Kollman charges, considering physiological pH [42]. Docking simulations were performed using AutoDock Vina, while AutoDock Tools 1.5.6 was used for ligand and protein preparation. For both enzymes, the docking lattice was centered on the ligand binding site and set with the following parameters: 30 × 30 × 30 Å with 1.000 Å spacing. Redocking with co-crystallized ligands and the calculation of Root Mean Square Deviation (RMSD) values using PyMOL were employed as validation steps for the docking protocol [43]. Ligand–protein interactions and images were obtained using the BIOVIA Discovery Studio Visualizer software.

#### In Silico Pharmacokinetics and Toxicity Analyses

To assess the pharmacokinetics of the SeBZF**1** compound, computational analysis was conducted using the pkCSM-pharmacokinetics platform (http://biosig.unimelb.edu.au/pkcsm). The compound was designed using ChemDraw Ultra 12.0 and subsequently converted to a SMILE sequence using Open Babel GUI. ADMET (Absorption, Distribution, Metabolism, Excretion, and Toxicity) data were evaluated: intestinal absorption, and distribution, with assessments of permeability parameters in the CNS and blood–brain barrier (BBB) and total clearance to analyze compound excretion. Regarding toxicity parameters, oral rat acute toxicity by lethal dose, 50% (LD50), oral rat subchronic toxicity by lowest-observed-adverse-effect (LOAEL) dose, AMES, and hepatotoxicity were evaluated.

### Statistical Analysis

Individual animals were used as experimental units. The results of the experiments were analyzed by the software GraphPad Prism (version 8.0.2) and expressed as the mean ± standard error of the mean (S.E.M.). The D'Agostino–Pearson normality test was applied to determine whether the data were normally distributed, with normality assessed separately for each group. Data transformation (square root) for non-parametric variables normalization was applied when necessary, using Excel, and subsequently normalized data were tested. Specifically, this transformation was used for latency data in the 7-NI, methylene blue, L-NAME, and sildenafil experiments, as well as for the crossing parameter in the ODQ experiment. The parametric data were analyzed by two-way analyses of variance (ANOVA), along with Tukey’s multiple comparisons post hoc test, or *t* test unpaired. Probability values less than p < 0.05 were considered significant. Although multiple behavioral endpoints were analyzed at the same test, each reflects distinct aspects of the behavioral phenotype and were therefore treated as an independent outcome.

## Results

### Involvement of the L-ARG-NO Pathway in the Acute Antidepressant-like Action of SeBZF1

Figure 2 shows the effects of the pretreatment of male mice with L-ARG (a precursor of NO formation), L-NAME (a non-selective NOS inhibitor), and 7-NI (a selective nNOS inhibitor) on the acute antidepressant-like action of SeBZF**1**.

Treatment with L-ARG (750 mg/kg) prevented the anti-immobility effects of SeBZF**1** (50 mg/kg, an effective dose) in the TST, as evidenced by changes in the latency to the first immobility episode (Fig. 2D) [F_(1,28)_ = 54.34; p < 0.0001] and the total immobility time (Fig. 2E) [F_(1,28)_ = 19.85; p = 0.0001]. Calculated values for Cohen’s *d* and effect size *r* were also obtained for latency time in the TST**:** SeBZF**1** 50 mg/kg × control group, Cohen’s *d* = −3.678 and effect size *r* = −0.878; L-ARG 750 mg/kg × control group, Cohen’s *d* = −0.950 and effect size *r* = −0.429; and interaction × control group, Cohen’s *d* = 0.042 and effect size *r* = 0.021. For immobility time, the calculated values were: SeBZF**1** 50 mg/kg × control group, Cohen’s *d* = 2.245 and effect size *r* = 0.747; L-ARG 750 mg/kg × control group, Cohen’s *d* = 0.269 and effect size *r* = 0.136; and interaction × control group, Cohen’s *d* = −0.083 and effect size *r* = −0.041.

The results depicted in Figs. 2F and 2G represent the effects from the co-administration of L-NAME (10 mg/kg) and a subeffective dose of SeBZF**1** (1 mg/kg) in the TST. Statistical differences were detected among the groups regarding the behavioral parameters evaluated, since an interaction between the analyzed factors was observed for latency [F_(1,32)_ = 7.00; p = 0.0125] and total immobility time [F_(1,32)_ = 4.88; p = 0.0345]. Further analyses showed that the administration of L-NAME with the subeffective dose of SeBZF**1** produced a synergistic effect on depressive-like behavior in the TST (p = 0.0003 and p = 0.0089 for latency and immobility time, respectively). Calculated values for Cohen’s *d* and effect size *r* were also obtained for latency time in the TST**:** SeBZF**1** 1 mg/kg × control group, Cohen’s *d* = −0.197 and effect size *r* = −0.098; L-NAME 10 mg/kg × control group, Cohen’s *d* = −0.317 and effect size *r* = −0.157; and interaction × control group, Cohen’s *d* = −2.381 and effect size *r* = −0.766. For immobility time, the calculated values were: SeBZF**1** 1 mg/kg × control group, Cohen’s *d* = 0.0411 and effect size *r* = 0.020; L-NAME 10 mg/kg × control group, Cohen’s *d* = 0.090 and effect size *r* = 0.045; and interaction × control group, Cohen’s *d* = 1.863 and effect size *r* = 0.682.

Figures 2H and 2I show the behavioral results of the co-administration of 7-NI (30 mg/kg) and a subeffective dose of SeBZF**1** (1 mg/kg). An interaction between 7-NI and SeBZF**1** was found when latency time [F_(1,33)_ = 71.25; p < 0.0001] and total immobility time [F_(1,33)_ = 12.63; p = 0.0012]. A synergistic effect between 7-NI and SeBZF**1** was observed in the TST, as latency increased (p < 0.0001) and immobility time decreased (p = 0.0001) following combined treatment. Calculated values for Cohen’s *d* and effect size *r* were also obtained for latency time in the TST**:** SeBZF**1** 1 mg/kg × control group, Cohen’s *d* = 0.102 and effect size *r* = 0.051; 7-NI 30 mg/kg × control group, Cohen’s *d* = 0.556 and effect size *r* = 0.267; and interaction × control group, Cohen’s *d* = −5.375 and effect size *r* = −0.937. For immobility time, the calculated values were: SeBZF**1** 1 mg/kg × control group, Cohen’s *d* = −0.060 and effect size *r* = −0.030; 7-NI 30 mg/kg × control group, Cohen’s *d* = 0.066 and effect size *r* = 0.033; and interaction × control group, Cohen’s *d* = 3.408 and effect size *r* = 0.862.

Table S1 shows that none of the treatments altered locomotor and exploratory activities in the OFT (p > 0.05).

### Involvement of cGMP in the Acute Antidepressant-like Action of SeBZF1

Figure 3 shows the effects of pretreatment of male mice with methylene blue (an inhibitor of NOS and sGC), ODQ (a sGC inhibitor), and sildenafil (a selective PDE-5 inhibitor) on the acute antidepressant-like action of SeBZF**1**.

Figures 3D and 3E illustrate the effects of co-administration of subeffective doses of methylene blue (10 mg/kg) with SeBZF**1** (1 mg/kg). Analysis showed significant differences between groups for latency time [F_(1,32)_ = 7.02; p = 0.0124] and total immobility time [F_(1,32)_ = 4.91; p = 0.0340]. Co-administration of methylene blue and the subeffective doses of the compound produced a synergistic effect in the TST as indicated by increased latency (p = 0.0010) and reduced immobility time (p = 0.0317). Calculated values for Cohen’s *d* and effect size *r* were also obtained for latency time in the TST**:** SeBZF**1** 1 mg/kg × control group, Cohen’s *d* = −0.121 and effect size *r* = −0.060; methylene blue 10 mg/kg × control group, Cohen’s *d* = −0.231 and effect size *r* = −0.115; and interaction × control group, Cohen’s *d* = −1.562 and effect size *r* = −0.616. For immobility time, the calculated values were: SeBZF**1** 1 mg/kg × control group, Cohen’s *d* = −0.152 and effect size *r* = −0.076; methylene blue 10 mg/kg × control group, Cohen’s *d* = 0.027 and effect size *r* = 0.013; and interaction × control group, Cohen’s *d* = 1.503 and effect size *r* = 0.600.

The effect of the combined administration of subeffective doses of ODQ (30 pmol/site) and SeBZF**1** (1 mg/kg) is shown in Figs. 3F and 3G. Significant differences were found for latency [F_(1,32)_ = 4.51; p = 0.0415] and total immobility time [F_(1,32)_ = 7.45; p = 0.0102]. Combined administration of subeffective doses was able to induce a decrease in total immobility time (p = 0.0455) but did not alter the latency to the first immobility episode (p > 0.05). Calculated values for Cohen’s *d* and effect size *r* were also obtained for latency time in the TST**:** SeBZF**1** 1 mg/kg × control group, Cohen’s *d* = 0.399 and effect size *r* = 0.195; ODQ 30 pmol/site × control group, Cohen’s *d* = 0.605 and effect size *r* = 0.289; and interaction × control group, Cohen’s *d* = −0.476 and effect size *r* = −0.231. For immobility time, the calculated values were: SeBZF**1** 1 mg/kg × control group, Cohen’s *d* = −0.061 and effect size *r* = −0.031; ODQ 30 pmol/site × control group, Cohen’s *d* = 0.076 and effect size *r* = 0.038; and interaction × control group, Cohen’s *d* = 1.361 and effect size *r* = 0.563.

In a parallel experiment, Figs. 3H and 3I show the effects of treatment with sildenafil (5 mg/kg) and SeBZF**1** (50 mg/kg, an effective dose) on latency time [F_(1,35)_ = 15.89; p = 0.0003] and total immobility time [F_(1,35)_ = 33.99; p < 0.0001]. The increase in latency (p = 0.0002) and decrease in total immobility time (p < 0.0001) induced by SeBZF**1** were blocked by sildenafil administration. Calculated values for Cohen’s *d* and effect size *r* were also obtained for latency time in the TST**:** SeBZF**1** 50 mg/kg × control group, Cohen’s *d* = −2.469 and effect size *r* = −0.777; sildenafil 5 mg/kg × control group, Cohen’s *d* = −0.205 and effect size *r* = −0.102; and interaction × control group, Cohen’s *d* = −0.722 and effect size *r* = −0.339. For immobility time, the calculated values were: SeBZF**1** 50 mg/kg × control group, Cohen’s *d* = 2.739 and effect size *r* = 0.807; sildenafil 5 mg/kg × control group, Cohen’s *d* = 0.619 and effect size *r* = 0.296; and interaction × control group, Cohen’s *d* = −0.154 and effect size *r* = −0.077.

Table S2 shows that none of the treatments altered locomotor and exploratory activity in the OFT (p > 0.05).

### Antidepressant‑Like Action of Subchronic Treatment with SeBZF1

Figures 4B and 4 C depict the behavioral outcomes of subchronic administration of a low dose of SeBZF**1** (1 mg/kg, i.g.). The unpaired *t* test revealed a significant effect of treatment on both latency [t(18) = 3.726, p = 0.0015)] and total immobility time [t(18) = 5.339, p < 0.0001]. Calculated values for Cohen’s *d* and effect size *r* were also obtained for latency time in the TST**:** SeBZF**1** 1 mg/kg × control group, Cohen’s *d* = 1.756 and effect size *r* = 0.659; For immobility time, the calculated values were: SeBZF**1** 1 mg/kg × control group, Cohen’s *d* = 2.517 and effect size *r* = 0.783.

Table S3 indicates that none of the treatments altered locomotor and exploratory activity in the OFT (p > 0.05).

### Acute and Subchronic SeBZF1 Treatment Decreased Brain NO_x_ Levels

**Fig. 5:**
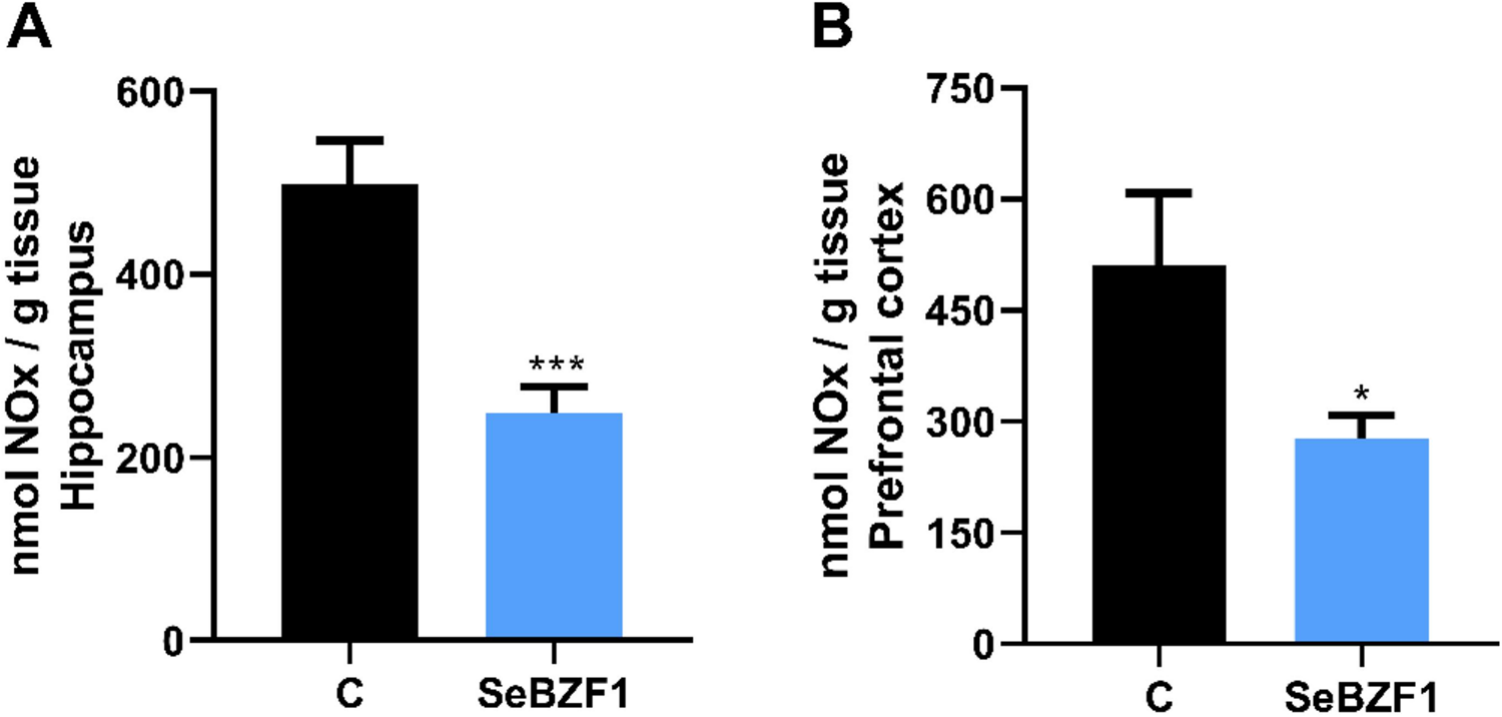
Effects of the acute SeBZF**1** (50 mg/kg, i.g.) treatment on NO_x_ levels in hippocampus (Fig.  5 A) and prefrontal cortex of mice (Fig. 5B). Values are expressed as the mean ± S.E.M. (n = 8–9 animals/group). *p < 0.05 and ***p < 0.001 compared with the control group. An unpaired *t* test was used for group comparisons

Figure 5 displays the results regarding determining NO_x_ levels following acute (50 mg/kg) treatment with SeBZF**1**. The unpaired *t* test indicated that compound reduced NO_x_ levels in the hippocampus (Fig. 5A) [t(15) = 4.545, p = 0.0004] and prefrontal cortex (Fig. 5B) [t(15) = 2.158, p = 0.0475]. Calculated values for Cohen’s *d* and effect size *r* were also obtained for NO_x_ levels in the hippocampus and cortex: for the hippocampus, SeBZF**1** 50 mg/kg × control group, Cohen’s *d* = 2.347 and effect size *r* = 0.761; for the cortex, SeBZF**1** 50 mg/kg × control group, Cohen’s *d* = 1.114 and effect size *r* = 0.487.

**Fig. 6:**
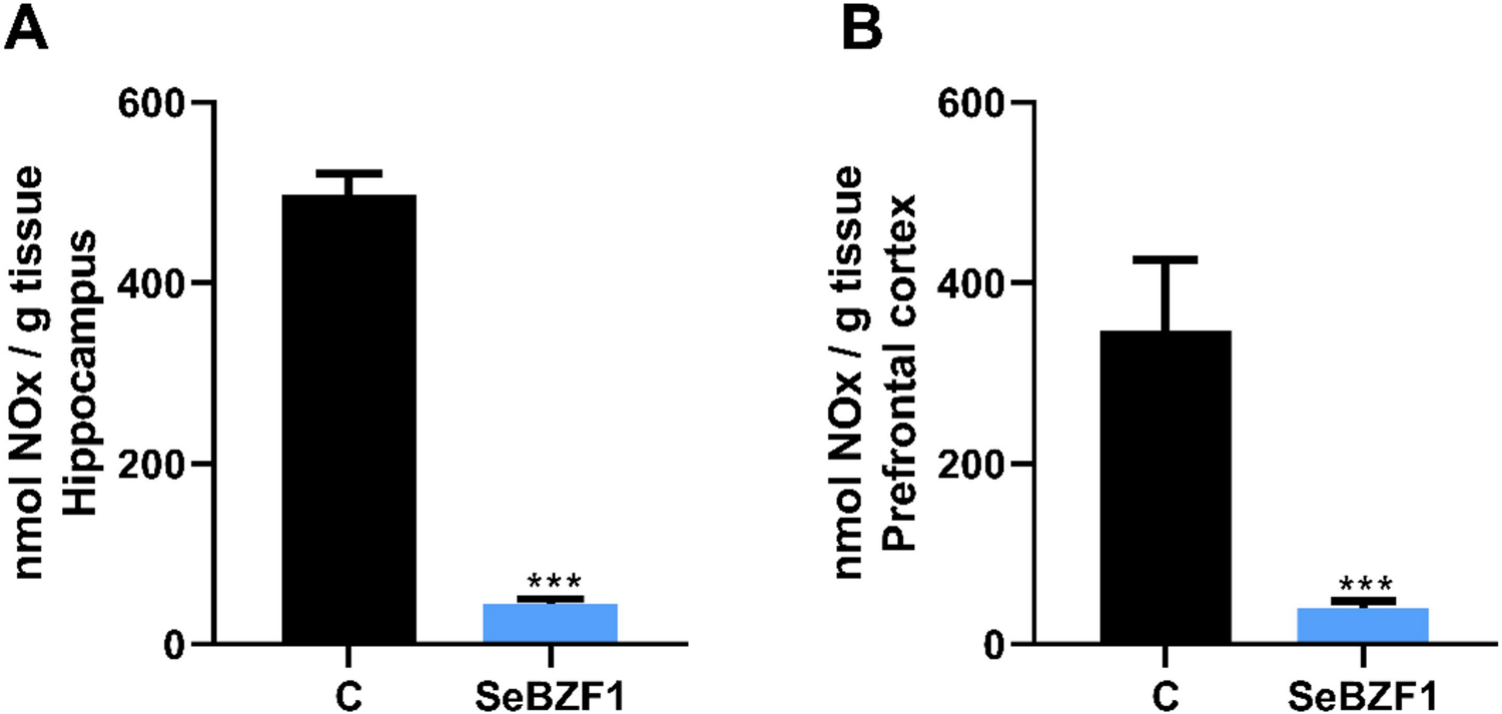
Effect of subchronic SeBZF**1** (1 mg/kg, i.g.) treatment on hippocampal (6A) and prefrontal (6B) cortex nitrite/nitrate (NO_x_) levels. Values are expressed as the mean ± S.E.M. (n = 10 animals/group). ***p < 0.001 compared to the control group. An unpaired *t* test was used for group comparisons

The results of NO_x_ level assessment after subchronic administration of SeBZF**1** (1 mg/kg) are shown in Fig. 6. The unpaired *t* test findings that the compound decreased NO_x_ levels in the hippocampus (Fig. 6A) [t(15) = 19.90, p < 0.0001] and prefrontal cortex (Fig. 6B) [t(15) = 17.25, p < 0.0001]. Calculated values for Cohen’s *d* and effect size *r* were also obtained for NO_x_ levels in the hippocampus and cortex: for the hippocampus, SeBZF**1** 1 mg/kg × control group, Cohen’s *d* = 10.270 and effect size *r* = 0.980; for the cortex, SeBZF**1** 1 mg/kg × control group, Cohen’s *d* = 8.900 and effect size *r* = 0.978.

### Subchronic Treatment with SeBZF1 Did Not Affect Markers of Systemic Toxicity

**Fig. 7:**
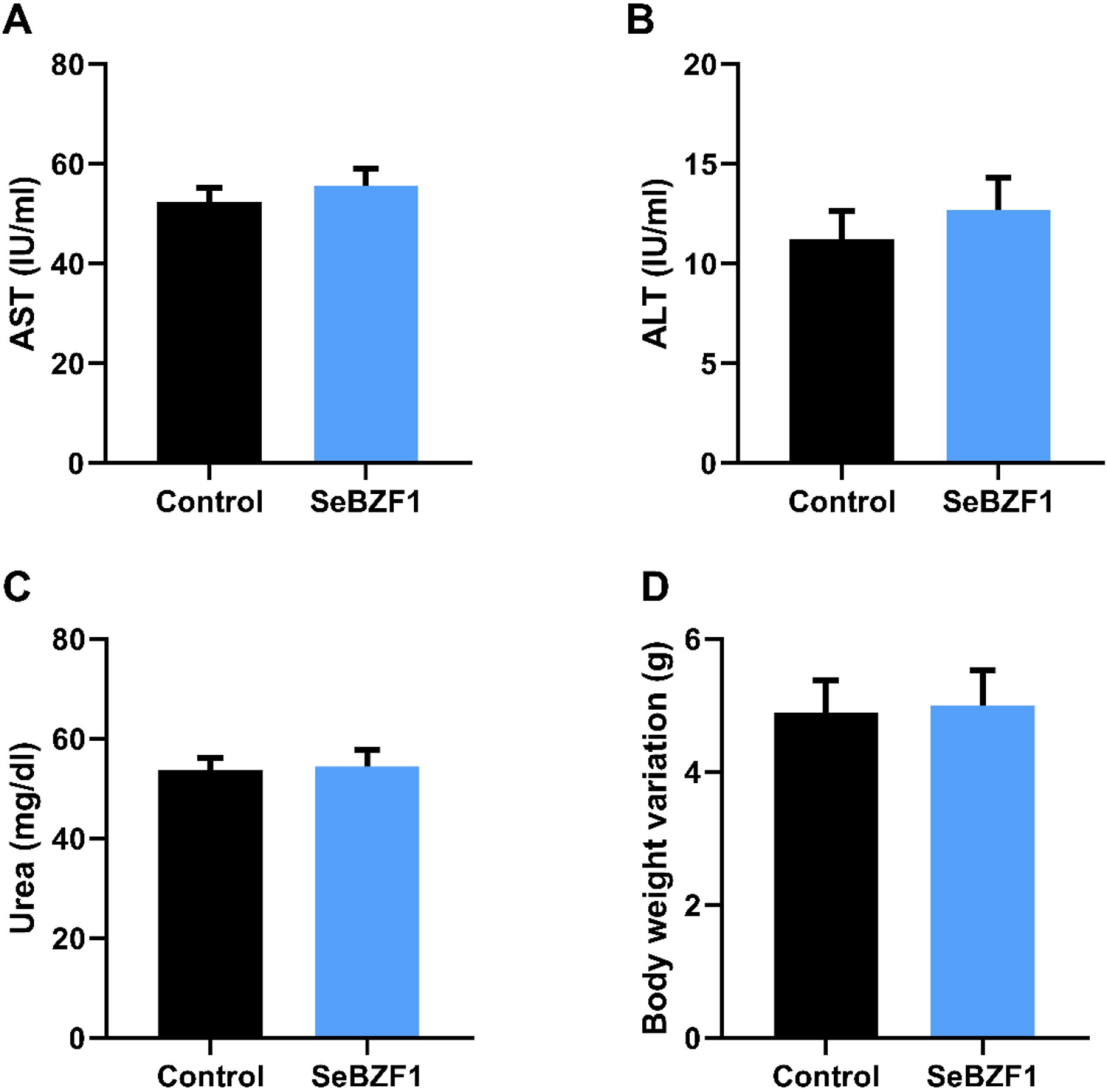
Effects of subchronic treatment with SeBZF**1** (1 mg/kg; i.g., 35 days) on liver and kidney toxicity markers and body weight variation in mice. No significant differences were observed between groups for AST activity [t(15) = 0.7222, p = 0.4814] (**A**), ALT activity [t(15) = 0.6707, p = 0.5126] (**B**), urea levels [t(15) = 0.1572, p = 0.8772] (**C**), or body weight gain [t(15) = 0.1544, p = 0.8793] (**D**). Data are expressed as mean ± SEM and analyzed using an unpaired *t* test

Figure 7 illustrates the results of liver and kidney toxicity tests, as well as the body weight variation of the animals, following daily administration of SeBZF**1** (1 mg/kg) for 35 days. The unpaired *t* test revealed that animals treated with SeBZF**1** did not exhibit significant changes in plasma AST activity [t(15) = 0.7222, p = 0.4814] (Fig. 7A) ALT activity [t(15) = 0.6707, p = 0.5126] (Fig. 7B), or plasma urea levels [t(15) = 0.1572, p = 0.8772] (Fig. 7C) compared with the control group. Furthermore, no difference was observed in body weight gain between animals treated with SeBZF**1** and controls [t(15) = 0.1544, p = 0.8793] (Fig. 7D). Calculated values for Cohen’s *d* and effect size *r* were also obtained for AST, ALT, urea, and body weight variation: for AST, SeBZF**1** 1 mg/kg × control group, Cohen’s *d* = 0.373 and effect size *r* = 0.183; for ALT, SeBZF**1** 1 mg/kg × control group, Cohen’s *d* = 0.346 and effect size *r* = 0.171; for urea, SeBZF**1** 1 mg/kg × control group, Cohen’s *d* = 0.081 and effect size *r* = 0.040; and for body weight variation, SeBZF**1** 1 mg/kg × control group, Cohen’s *d* = 0.079 and effect size *r* = 0.039.

### Possible Interactions of SeBZF1 with iNOS and nNOS Enzymes

**Fig. 8:**
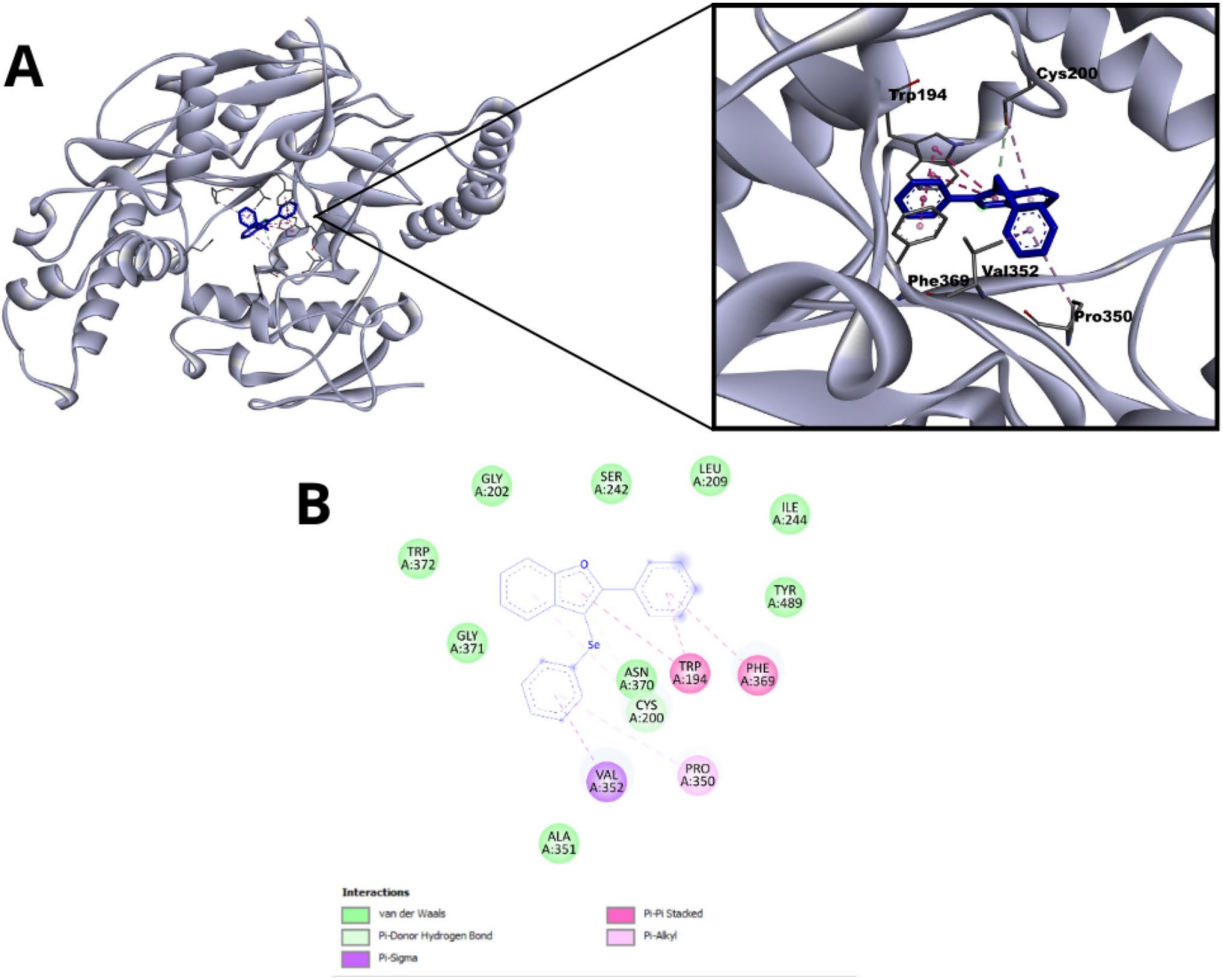
Protein–ligand interaction predicted by molecular docking for SeBZF**1** binding to iNOS (**A**) and 2D interacting residues (**B**)

Figure 8A presents the preferred binding conformations of SeBZF**1** in the catalytic site of iNOS. Molecular docking studies revealed a high affinity of SeBZF**1** for the crystallographic structure of iNOS, with a docking score of −10.3 kcal/mol. The results shown in Fig. 8B indicate that SeBZF**1** interacts with the Cis200 residue through a π-donor hydrogen bond. In addition, a π-sigma interaction was observed with the Val352 residue and a π-alkyl interaction with the Pro350 residue. SeBZF**1** also forms π-π interactions with Trp194 and Phe369 through the aromatic ring directly attached to the benzofuran core.

**Fig. 9:**
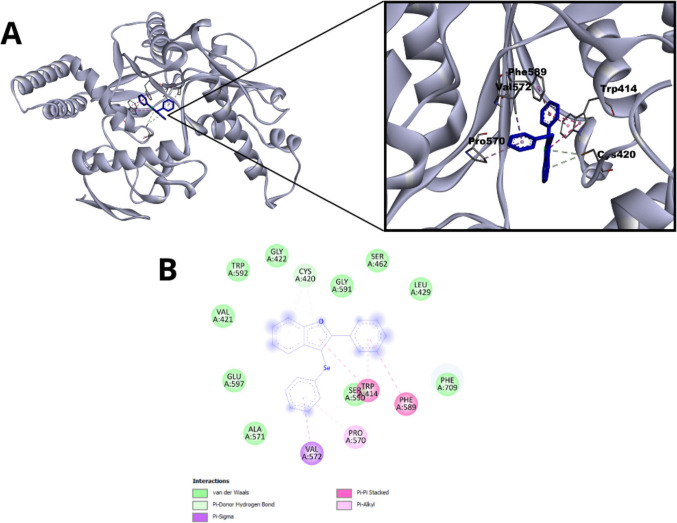
Predicted analysis of protein–ligand interaction through molecular docking for SeBZF**1** binding to nNOS (**A**) and (**B**) 2D interacting residues

Figure 9A shows the preferred binding conformations of SeBZF**1** in the catalytic site of nNOS. Molecular docking studies revealed a significant affinity of SeBZF**1** for the crystallographic structure of nNOS, with a docking score of −9.4 kcal/mol. The results illustrated in Fig. 9B indicate that SeBZF**1** exhibits a π-alkyl interaction with Pro570 residue, a π-sigma interaction with Val572 residue, a π-π interaction with the Trp414 and Phe589 residues, a π-donor hydrogen bond with the Cys420 residue.

To validate the docking protocol, a re-docking procedure was performed using the co-crystallized ligands of iNOS and nNOS. The root-mean-square deviation (RMSD) values between the predicted and experimental poses were 1.078 Å and 1.694 Å (Figure S1A and S2B), respectively, indicating good agreement and reliability of the docking protocol.

To provide a comparative framework for the docking results, known NOS inhibitors were included as reference compounds. The preferred binding conformations of 1400 W for iNOS (Figure S2) and 7-NI for nNOS (Figure S3) within the catalytic sites. Both compounds exhibited high binding affinity toward their respective targets, with docking scores of − 6.976 kcal/mol for 1400 W and − 7.033 kcal/mol for 7-NI. These findings support the reliability of the docking protocol and provide a benchmark for comparison, as SeBZF**1** demonstrated a more favorable binding energy than the reference inhibitors.

### Computational Prediction of the Pharmacokinetic Profile and Toxicity of SeBZF1

Table 1 represents the pharmacokinetic and toxicity parameters found for SeBZF**1** using ADMET analysis. The data demonstrated that intestinal absorption of the compound is predicted to be high. The assessments of permeability and distribution parameters in the CNS and BBB suggest that SeBZF**1** is potentially able to penetrate the CNS through the BBB. Regarding excretion, the compound exhibits a relatively high total clearance value. In addition, toxicity parameters showed SeBZF**1** is predicted to be non-mutagenic (negative AMES test) and non-hepatotoxic, with an LD_50_ of 1.871 mol/kg. The lowest observed adverse effect level (LOAEL) dose was predicted to be 0.134 mg/kg_bw/day.

**Table 1:** Computational prediction of pharmacokinetic and toxicity parameters (ADMET) for SeBZF**1**

Properties	Parameters	SeBZF**1**
Absorption	Intestinal absorption	100% absorbed
Distribution	CNS permeability	−1.06 log PS
	BBB permeability	0.98 log BB
Excretion	Total clearance	1.984 log ml/min/kg
Toxicity	AMES toxicity	No
	LD_50_	1.871 mol/kg
	LOAEL	0.134 mg/kg_bw/day
	Hepatotoxicity	No

(CNS: central nervous system; BBB: blood–brain barrier; LD_50_: lethal dose, 50%; LOAEL: lowest-observed-adverse-effect).

## Discussion

The present study demonstrated that SeBZF**1** produces significant antidepressant-like effects in the TST, involving modulation of the nitrergic pathway. Pretreatment with L-ARG inhibited the decrease in immobility time elicited by acute SeBZF**1** administration. At the same time, co-administration of subeffective acute doses of NOS, sGC, and PDE-5 enzyme inhibitors in combination with SeBZF**1** produced antidepressant-like effects. In addition, NO_x_ levels were reduced in brain structures after acute and subchronic exposure to SeBZF**1**, without signs of apparent toxicity, according to experimental data corroborated by computational analyses. Moreover, molecular docking analysis revealed a relevant binding affinity of SeBZF**1** for the catalytic sites of both nNOS and iNOS enzymes, supporting its potential to modulate NO synthesis. Although SeBZF**1** has been previously reported to exert antidepressant-like effects through monoaminergic [19] and glutamatergic [21] modulation, the present study advances current knowledge by providing integrated mechanistic evidence supporting the involvement of the nitrergic pathway. Importantly, this is supported not only by pharmacological interaction studies, but also by biochemical findings (reduced NO_x_ levels in brain regions) and molecular docking analyses, which together offer a multilevel understanding of the compound mechanism of action. In the present study, only one behavioral paradigm was employed. However, the antidepressant-like effects of SeBZF**1** in the FST have been previously demonstrated [19], supporting the robustness of its behavioral profile. In this context, the TST was selected due to its lower variability and reduced susceptibility to confounding factors, such as hypothermia and physical fatigue, which may influence performance in the FST [44, 45].

Several studies have linked an increase in L-ARG, a NO precursor, to depressive-like behavior [42, 46]. In our study, pretreatment with L-ARG significantly blocked the antidepressant-like effects of SeBZF**1**, consistent with findings that L-ARG blocks the effects of antidepressants such as bupropion, venlafaxine, and imipramine [46–48]. These effects likely involve NO-mediated effects on serotonergic and dopaminergic systems [5, 49]. This is consistent with previous findings showing that the dopaminergic pathway via D_1_ and D_2_ receptors [20], the serotonergic pathway via 5-HT_1A_ and 5-HT_2A/C_ receptors [19], and MAO inhibition [23], are also involved in its antidepressant-like action in Swiss mice.

In addition to monoaminergic involvement, we previously demonstrated that SeBZF**1** also exerts antidepressant-like effects through direct or indirect modulation of the glutamatergic system [21]. NMDA receptors are mainly located in the limbic system, a brain region closely associated with depression and other psychiatric disorders. Several studies demonstrate that treatment with ketamine, a noncompetitive NMDA receptor antagonist, produces a rapid antidepressant response following a single low dose, even in treatment-resistant depression patients [50–52]. This rapid onset and potential for improved adherence are highly desirable features in the development of new antidepressant therapies.

The release of glutamate and activation of NMDA receptors lead to an influx of calcium (Ca^2+^), increasing the intracellular concentration of this ion. Ca^2^⁺ then binds to a cytoplasmic protein, forming a Ca^2^⁺/calmodulin complex that activates NOS, thereby increasing NO levels [53, 54]. Due to the brain's vulnerability to nitrosative damage, structures associated with depression, such as the hippocampus and amygdala, are also linked to cognitive decline. This vulnerability may predispose patients to developing depression [55].

In this context, the present results demonstrated that co-administration of subeffective doses of SeBZF**1** and NOS inhibitors, L-NAME, or 7-NI, produced synergistic antidepressant-like effects in the TST. These results indicate that inhibition of NO synthesis may be directly or indirectly implicated in the compound’s antidepressant-like effects. Together with previous data, our findings suggest that the antidepressant-like effects of SeBZF**1** involve a direct or apparent antagonism toward the NMDA receptor NO-cGMP pathway, which appears to play an important role in its mechanism of action.

Building upon these findings and to further elucidate the involvement of the nitrergic signaling pathway, the present study investigated the effects of a subeffective dose of ODQ, a potent and selective inhibitor of sGC [56], when co-administered with a subeffective dose of SeBZF**1**. The results showed that this co-administration produced an antidepressant-like effect. Interestingly, the combination of ODQ and SeBZF**1** reduced immobility time without affecting latency to the first immobility episode. These parameters reflect distinct aspects of behavioral response, with latency associated with the initial coping strategy and immobility representing the maintenance of a depressive-like state. Thus, this pattern suggests that the treatment primarily influences the persistence of depressive-like behavior rather than the initial response to stress [57]. In another experiment, simultaneous administration of subeffective doses of methylene blue, an inhibitor of NOS and sGC [58], and SeBZF**1** also produced an antidepressant-like response in the TST. Furthermore, sildenafil, a selective PDE-5 inhibitor [59], prevented the anti-immobility effects of SeBZF**1** in the TST. The function of nNOS is mainly mediated by the sGC pathway, which is activated by cGMP and affects postsynaptic neuronal excitability [5]. Corroborating our results, previous studies suggest that NO production, and consequently increased serum NO levels, can occur in depression, whereas NOS-inhibiting medications contribute to improving mood disorders [5, 60]. In the brain, nNOS is concentrated in many areas associated with depression, including the hypothalamus, locus coeruleus, and hippocampus. Indeed, numerous studies have reported that NOS inhibitors, depending on their concentration, display antidepressant-like properties [9, 49, 61]. Despite behavioral evidence suggesting modulation of the cGMP pathway, a limitation of this study is the lack of direct measurement of cGMP levels.

An important point to consider, in light of the effects observed with sildenafil, is that its actions are not limited to PDE-5 inhibition. Sildenafil has been reported to exert neuroprotective and vascular effects [62], suggesting that its influence on behavioral responses may not be restricted to cGMP modulation, but may also involve changes in cerebral perfusion and neuronal viability. Moreover, sildenafil can cross the BBB and has been associated with dopaminergic modulation, including effects on neurogenesis, memory, and depressive-like symptoms [63]. In parallel, SeBZF**1** has also been shown to act on the dopaminergic pathway. Therefore, the interaction between sildenafil and SeBZF**1** likely extends beyond the NO–cGMP pathway, potentially involving dopaminergic mechanisms, which may contribute to the observed reversal of the antidepressant-like effect.

Organoselenium compounds have been increasingly investigated for their potential antidepressant-like effects. Among them, Ebselen is one of the most extensively studied candidates, with reported actions involving antioxidant properties, modulation of glutamatergic signaling, and others [24, 25]. Both behavioral and structural aspects may contribute to the distinct profile of SeBZF**1** compared to Ebselen. Although Ebselen has shown limited or variable effects in the TST, a sensitive and predictive model for antidepressant-like activity [57], SeBZF**1** significantly reduced immobility time in this test Additionally, SeBZF**1** contains a benzofuran core, a scaffold associated with antidepressant-like effects and present in drugs such as citalopram [64] and escitalopram [65], which may contribute to its pharmacological profile. In this context, canola oil has been widely used in studies involving organoselenium compounds by different research groups due to the lipophilic nature of these compounds [24, 25]. Notably, canola oil contains omega-3 polyunsaturated fatty acids, which have been associated with antidepressant-like effects in preclinical studies [66]. However, all experimental groups, including control and induced groups, received the same vehicle in the present study, thereby minimizing its potential confounding effect on group comparisons.

The hippocampus and prefrontal cortex were specifically examined in this study, as they are brain structures strongly associated with depression. The enzyme nNOS, for example, is predominantly expressed by neurons in these regions, leading to an increase in NO production, which may contribute to the onset of depression symptoms [49, 67]. Reinforcing our hypothesis regarding the involvement of the NO pathway in the antidepressant effect of SeBZF**1**, our study also revealed that both acute and subchronic administrations of the compound were able to reduce NO levels in the hippocampus and prefrontal cortex of animals. In fact, other studies have previously linked the antidepressant action of clinically used compounds, such as fluoxetine and imipramine, to this same pathway [47, 68].

The molecular docking results corroborated the pharmacological findings described above as the analysis revealed a favorable interaction between the SeBZF**1** compound and the nNOS and iNOS enzymes. The nNOS isoform is a constitutive enzyme activated by intracellular Ca^2+^, whereas iNOS expression is induced by pro-inflammatory cytokines or endotoxins and is activated independently of intracellular Ca^2+^ [49]. Docking analysis revealed a high binding affinity and a suitable conformation of the formed complex were observed [69, 70]. These results suggest that SeBZF**1** has the potential to bind and interact effectively with the target proteins, which may contribute to its biological activity. This hypothesis is consistent with the observed decrease in NO_x_ levels observed in the present study. Although NO_x_ levels and pharmacological approaches support the involvement of the nitrergic pathway in the antidepressant-like effects of SeBZF**1**, some limitations should be acknowledged. The absence of a direct assessment of NOS enzymatic activity, as well as the lack of experimental validation of enzyme–ligand binding, precludes distinguishing between direct inhibition and indirect modulation of the enzyme. Nevertheless, molecular docking analyses demonstrated relevant binding affinity of SeBZF**1** to the catalytic sites of NOS isoforms, providing structural support for a potential direct interaction with the enzyme. Although we focused on nNOS and iNOS due to their established involvement in neuroinflammation, a contribution of eNOS to the observed effects cannot be ruled out.

Previous studies with the compound SeBZF**1** demonstrated that it exerts an antidepressant-like action in male and female mice when administered repeatedly for 7 days [20]. In the present study, male mice were selected to reduce biological variability associated with hormonal fluctuations, allowing a more controlled investigation of the underlying mechanisms of action. To further investigate the subchronic antidepressant-like effects of the compound, animals received SeBZF**1** for 35 days. The low dose of 1 mg/kg was sufficient to reduce immobility time in the TST, with animals remaining more active during the test, indicating an antidepressant-like action. The chronicity of antidepressant treatment is crucial to the management of depressive disorders, as it ensures sustained symptom relief, prevents relapses, and promotes long-term recovery [71]. In the present study, antidepressant-like effects were observed at a higher dose following acute administration, whereas repeated (subchronic) treatment produced similar effects at a lower dose. This pattern may reflect cumulative pharmacological effects and neuroadaptive processes associated with repeated exposure. In addition, the lipophilic nature of SeBZF**1** may favor tissue distribution and accumulation over time, potentially contributing to enhanced efficacy under subchronic conditions. Clinical observations highlight that high dosages of therapeutic agents often correlate with undesirable outcomes, including sedative effects. Therefore, low doses become preferable due to the concomitant reduction in potential side effects [72, 73].

Depression treatment is often prolonged, and long-term antidepressant use may lead to adverse effects, including liver and kidney damage. Notably, drug-induced liver injury is a major cause of drug discontinuation during development or withdrawal after approval. Therefore, monitoring organ function is essential to prevent latent toxicity [74, 75]. In the present study, we assessed the potential toxicological effects of SeBZF**1** after 35 days of repeated oral administration (1 mg/kg). Liver function was evaluated by measuring serum levels of AST and ALT, while kidney function was assessed through urea levels. Additionally, the animals’ body weight was monitored throughout the treatment period, as changes in weight may reflect systemic toxicity or metabolic alterations [76, 77]. Notably, SeBZF**1** did not induce changes in body weight or affect biochemical markers of liver and kidney function. These findings suggest that SeBZF**1**, at a low dose, is well tolerated and appears safe for subchronic use, supporting its potential as a novel antidepressant candidate. However, we recognize that biochemical markers alone may not be sensitive enough to detect subtle tissue alterations, which would be more accurately evaluated through histopathological analysis.

While the ADMET-predicted LOAEL suggests a lower toxicity threshold than the dose of SeBZF**1** used in this study, this apparent discrepancy should be interpreted with caution. In silico models primarily estimate potential hazard based on chemical structure and may not fully capture compound-specific pharmacokinetic processes observed in vivo [78]. In addition, they do not provide detailed information on the biological pathways underlying toxicity and may be insufficient to accurately predict toxic effects. In this context, our experimental findings provide complementary, biologically relevant evidence supporting the safety of the administered dose.

Predicting the effects induced by a drug when administered orally, including gastrointestinal absorption, distribution, and excretion, is a crucial component of drug discovery [79]. Computational analyses indicated that SeBZF**1** has favorable pharmacokinetic properties, including predicted gastrointestinal absorption and the ability to cross the BBB, which is relevant for central nervous system activity. Compounds with logBB values greater than 0.3 are generally considered capable of BBB penetration [80]. Furthermore, AMES and hepatotoxicity predictions suggested a low risk of mutagenic and hepatic adverse effects, which is consistent with the absence of overt toxicity observed in vivo. Appropriate pharmacokinetic and toxicity profiles, together with efficacy, are crucial determinants of successful drug development [81].

When interpreted together with the experimental data, these predictions provide supportive, rather than definitive, insights into the pharmacological profile of SeBZF**1**. The predicted BBB permeability is consistent with the central behavioral effects observed, as well as with the modulation of NO-related pathways in brain regions such as the hippocampus and prefrontal cortex. Similarly, the predicted safety profile aligns with the absence of detectable toxicity following subchronic administration. Although ADMET predictions do not establish a direct pharmacokinetic–pharmacodynamic relationship, the favorable absorption and predicted BBB permeability of SeBZF**1** may help explain its efficacy under both acute high-dose and subchronic low-dose conditions. Efficient systemic exposure and central penetration could allow pharmacological activity even at lower doses following repeated administration, potentially reflecting cumulative effects or adaptive neurobiological responses commonly observed in chronic antidepressant paradigms. However, this interpretation requires confirmation through dedicated pharmacokinetic studies.

## Conclusions

In conclusion, we demonstrated a probable modulatory role of the nitrergic pathway in the antidepressant-like action of SeBZF**1** in male Swiss mice. As these targets have been increasingly associated with the pathophysiology of depression, the present results may contribute to the development of novel therapeutic strategies for this disorder. Additionally, the subchronic therapeutic efficacy and absence of toxicity signals observed in our study further underscore the potential clinical relevance of SeBZF**1**. Lastly, computational analyses indicate that the compound is a promising candidate for oral administration due to its favorable pharmacokinetic properties and lack of toxicity.

## Supplementary Information

Below is the link to the electronic supplementary material.

Supplementary file1 (DOCX 633 KB)

## Data Availability

The datasets generated and/or analyzed during the current study are available from the corresponding author upon request.
