# Supplementary material for: 2-Phenyl-3-(Phenylselanyl)Benzofuran As a Promising Antidepressant Candidate: Mechanistic Insights Into Nitrergic Modulation and Subchronic Efficacy and Safety Profiling
Source: Mol Neurobiol. 2026 Jul 16;63(1):771. doi: 10.1007/s12035-026-06058-6 (PMC13375843; doi:10.1007/s12035-026-06058-6)
Supplement: Supplementary file 1 — Supplementary file1 (DOCX 633 KB) [file 12035_2026_6058_MOESM1_ESM.docx]

**SUPPLEMENTARY MATERIAL**

**Characterization of the compound 2-phenyl-3-(phenylselanyl)benzo[*b*]furan (SeBZF1)**

****The compound was a white solid with a melting point of 40-42 ºC.

**^1^H NMR** (500 MHz, CDCl_3_) δ 8.20 (d, *J* = 7.2 Hz, 2H), 7.55 (d, *J* = 8.2 Hz, 1H), 7.51 (d, *J* = 7.8 Hz, 1H), 7.48 – 7.42 (m, 2H), 7.42 – 7.37 (m, 1H), 7.35 – 7.31 (m, 1H), 7.29 (dt, *J* = 7.8, 1.4 Hz, 2H), 7.23 (dd, *J* = 8.8, 6.1 Hz, 1H), 7.14 (t, *J* = 7.3 Hz, 3H). **^13^C NMR** (125 MHz, CDCl_3_) δ 157.2, 154.1, 131.9, 131.4, 130.1, 129.3, 129.3, 129.2, 129.2, 129.1, 128.4, 127.8, 126.2, 125.2, 123.4, 121.2, 111.2, 99.7, 77.25.

**Table S1.** Locomotor and exploratory activities of mice treated with SeBZF**1** and/or agents of the NO pathway in the OFT.

| Treatment protocol | Groups | Number of crossings | Number of rearings |
| --- | --- | --- | --- |
| L-ARG 750 mg/kg | Control | 65.25 ± 4.53 | 25.00 ± 2.83 |
|  | L-ARG | 75.63 ± 5.76 | 31.88 ± 2.88 |
|  | SeBZF**1** | 73.88 ± 7.07 | 22.75 ± 3.62 |
|  | L-ARG + SeBZF**1** | 77.75 ± 8.24 | 28.75 ± 4.62 |
| L-NAME 10 mg/kg | Control | 72.67 ± 4.16 | 22.89 ± 4.13 |
|  | L-NAME | 62.38 ± 8.13 | 18.11 ± 4.81 |
|  | SeBZF**1** | 77.10 ± 7.26 | 31.22 ± 4.38 |
|  | L-NAME + SeBZF**1** | 51.44 ± 3.48 | 15.20 ± 3.26 |
| 7-NI 25 mg/kg | Control | 71.78 ± 4.96 | 27.11 ± 4.00 |
|  | 7-NI | 75.00 ± 6.45 | 23.11 ± 4.86 |
|  | SeBZF**1** | 66.60 ± 6.48 | 29.10 ± 3.42 |
|  | 7-NI + SeBZF**1** | 69.44 ± 6.74 | 25.11 ± 3.76 |
|  |  |  |  |

The results represent the means ± S.E.M. of 8-10 mice/group. Data analyses were carried out through the Two-way ANOVA.

**Table S2.** Locomotor and exploratory activities of mice treated with SeBZF**1** and/or agents of the NO pathway in the OFT.

| Treatment protocol | Groups | Number of crossings | Number of rearings |
| --- | --- | --- | --- |
| MB 10 mg/kg | Control | 61.00 ± 5.57 | 34.67 ± 3.69 |
|  | MB | 54.33 ± 4.32 | 28.56 ± 3.10 |
|  | SeBZF**1** | 52.33 ± 4.62 | 26.44 ± 2.95 |
|  | MB + SeBZF**1** | 66.00 ± 5.13 | 31.22 ± 4.47 |
| Sildenafil 5 mg/kg | Control | 71.82 ± 13.44 | 24.73 ± 4.80 |
|  | Sildenafil | 60.50 ± 11.67 | 18.00 ± 4.37 |
|  | SeBZF**1** | 64.50 ± 11.22 | 23.50 ± 3.77 |
|  | Sildenafil + SeBZF**1** | 80.22 ± 15.46 | 21.89 ± 5.29 |
| ODQ 30 ρmol/site | Control | 88.50 ± 17.33 | 20.89 ± 3.72 |
|  | ODQ | 96.09 ± 10.68 | 32.60 ± 4.92 |
|  | SeBZF**1** | 68.22 ± 15.31 | 31.13 ± 7.57 |
|  | ODQ + SeBZF**1** | 87.67 ± 13.84 | 32.22 ± 5.81 |
|  |  |  |  |

The results represent the means ± S.E.M. of 8-10 mice/group. Data analyses were carried out through the Two-way ANOVA.

**Table S3.** Effects of the chronic treatment with SeBZF**1** on the OFT in male Swiss mice.

| Treatment protocol | Groups | Number of crossings | Number of rearings |
| --- | --- | --- | --- |
| Chronic exposure | Control x SeBZF**1** 1mg/kg | t=2.041, df=18 | t=1.927, df=18 |
|  |  |  |  |

**Figure S1**


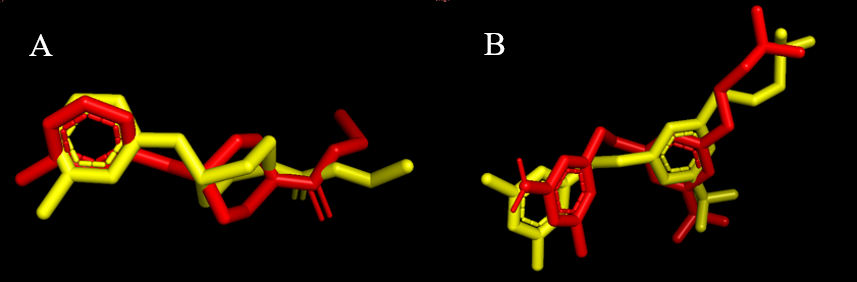


Validation of the docking protocol by re-docking analysis. Root-mean-square deviation (RMSD) values were calculated by comparing the predicted poses (red) with the co-crystallized reference ligands (yellow). (A) Re-docking for iNOS (PDB ID: 3E7G) yielded an RMSD of 1.078 Å; (B) re-docking for nNOS (PDB ID: 6AV2) yielded an RMSD of 1.694 Å.

**Figure S2**

**
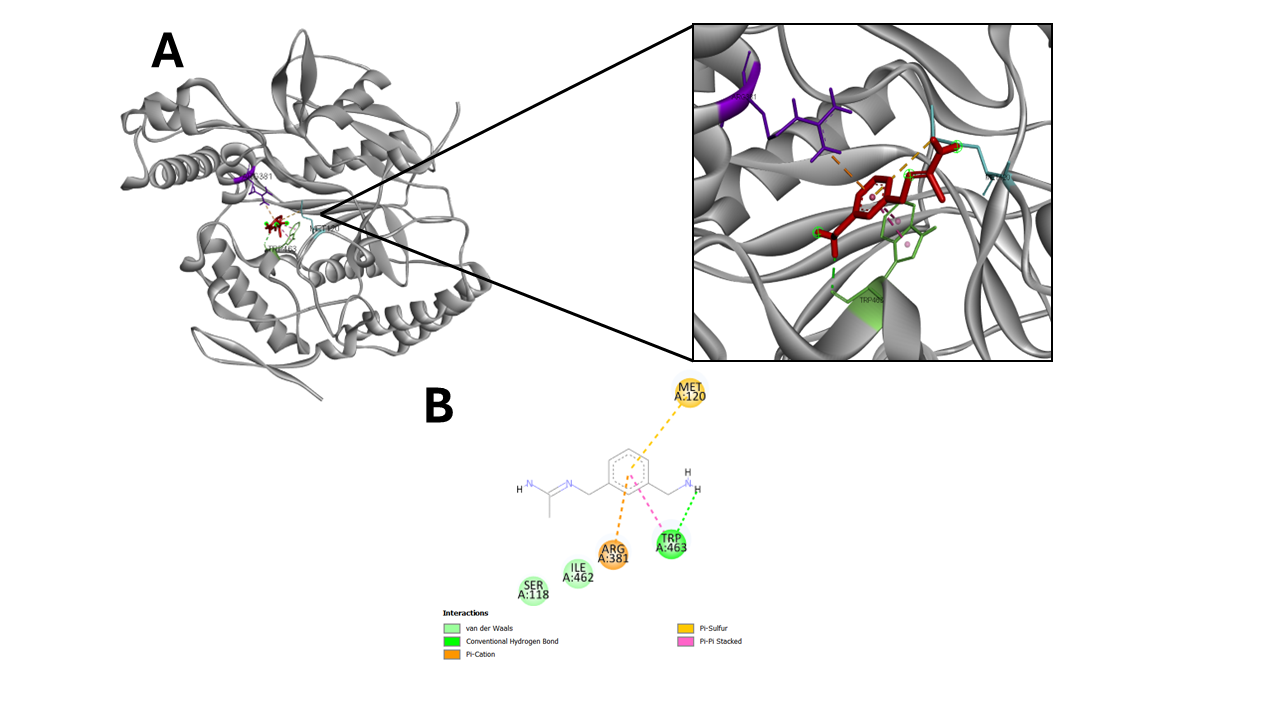
**

Protein-ligand interaction predicted by molecular docking for positive control (1400W) binding to iNOS (A) and 2D interacting residues (B).

**Figure S3**

**
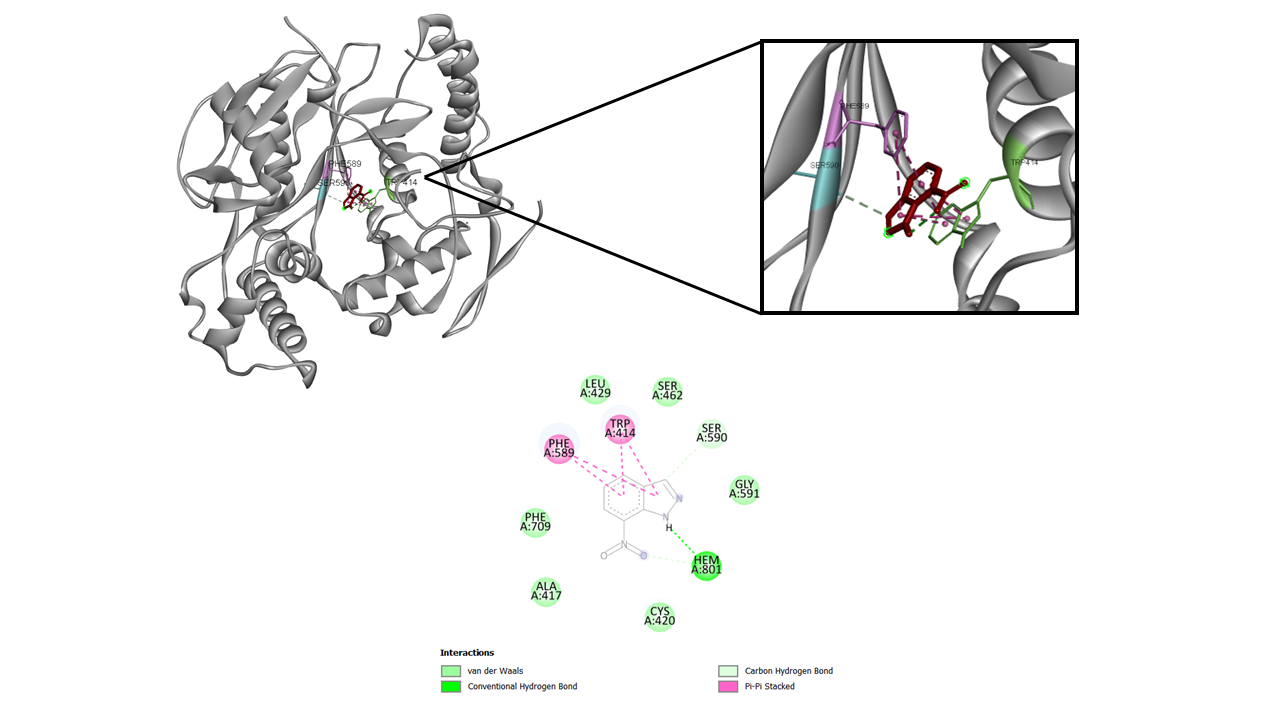
**

Protein-ligand interaction predicted by molecular docking for positive control (7-NI) binding to nNOS (A) and 2D interacting residues (B).
